# (*E*)-1-([1,1′-Biphen­yl]-4-yl)-2-(1,3,3-tri­methylindolin-2-yl­idene)ethanone

**DOI:** 10.1107/S1600536811045168

**Published:** 2011-11-09

**Authors:** Oscar F. Vázquez-Vuelvas, Armando Pineda-Contreras, David Morales-Morales, Simón Hernández-Ortega, Mikhail Tlenkopatchev

**Affiliations:** a Facultad de Ciencias Químicas, Universidad de Colima, km 9 Carr. Colima-Coquimatlán s/n, Coquimatlán, Colima 28400, Mexico; b Instituto de Química, Universidad Nacional Autónoma de México, Circuito Exterior, Ciudad Universitaria, México D.F. 04510, Mexico; c Instituto de Investigaciones en Materiales, Universidad Nacional Autónoma de México, Circuito Exterior, Ciudad Universitaria, México D.F. 04510, Mexico

## Abstract

The title compound, C_25_H_23_NO, consists of a biphenyl-4-carbonyl unit attached to an exocyclic double bond group at position 2 of an indole unit, which presents methyl groups as substituents at positions 1 and 3. The mol­ecular conformation is *s-cis* with an *E* configuration, supported by weak intra­molecular C—H⋯O contacts involving the methyl groups and the carbonyl function. The rings of the biphenyl group are twisted by 37.13 (5)°. In the crystal, C—H⋯O and C—H⋯π inter­actions link the molecules.

## Related literature

For background to the Fisher base (2,3-dihydro-1*H*-1,3,3-trimethyl-2-methyl­ene­indole), see: Minkin (2004 ▶); Przhiyalgovskaya *et al.* (1987 ▶). For applications of derivatives of the Fisher base in materials and organic synthesis, see: Corns *et al.* (2009 ▶); Shimkin *et al.* (2006 ▶); Song *et al.* (2005 ▶); Tarshits *et al.* (2005 ▶); Cui & Kim (2004 ▶).
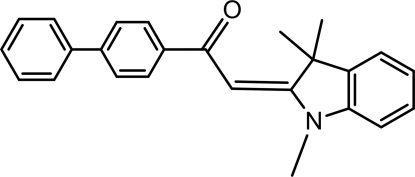


## Experimental

### 

#### Crystal data

C_25_H_23_NO
*M* *_r_* = 353.44
Monoclinic, 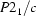

*a* = 12.3274 (13) Å
*b* = 15.7586 (16) Å
*c* = 10.3848 (11) Å
β = 104.719 (2)°
*V* = 1951.2 (4) Å^3^
*Z* = 4
Mo *K*α radiation
μ = 0.07 mm^−1^
*T* = 298 K
0.36 × 0.28 × 0.26 mm

#### Data collection

Bruker SMART APEX CCD area-detector diffractometer
15883 measured reflections
3584 independent reflections
2585 reflections with *I* > 2σ(*I*)
*R*_int_ = 0.033

#### Refinement

*R*[*F* ^2^ > 2σ(*F* ^2^)] = 0.038
*wR*(*F* ^2^) = 0.106
*S* = 0.97
3584 reflections
247 parameters
H-atom parameters constrained
Δρ_max_ = 0.18 e Å^−3^
Δρ_min_ = −0.18 e Å^−3^

### 

Data collection: *SMART* (Bruker, 1999 ▶); cell refinement: *SAINT* (Bruker, 1999 ▶); data reduction: *SAINT*; program(s) used to solve structure: *SHELXS97* (Sheldrick, 2008 ▶); program(s) used to refine structure: *SHELXL97* (Sheldrick, 2008 ▶); molecular graphics: *SHELXTL* (Sheldrick, 2008 ▶) and *DIAMOND* (Brandenburg, 2002 ▶); software used to prepare material for publication: *SHELXTL*.

## Supplementary Material

Crystal structure: contains datablock(s) I, global. DOI: 10.1107/S1600536811045168/bh2373sup1.cif

Structure factors: contains datablock(s) I. DOI: 10.1107/S1600536811045168/bh2373Isup2.hkl

Supplementary material file. DOI: 10.1107/S1600536811045168/bh2373Isup3.cml

Additional supplementary materials:  crystallographic information; 3D view; checkCIF report

## Figures and Tables

**Table 1:** Hydrogen-bond geometry (Å, °) *Cg*1, *Cg*2 and *Cg*3 are the centroids of the C21–C26, C12–C17 and C4–C9 rings, respectively.

*D*—H⋯*A*	*D*—H	H⋯*A*	*D*⋯*A*	*D*—H⋯*A*
C19—H19*B*⋯O1	0.96	2.36	3.112 (2)	135
C20—H20*B*⋯O1	0.96	2.31	3.078 (2)	137
C24—H24⋯O1^i^	0.93	2.54	3.465 (2)	171
C7—H7⋯*Cg*1^ii^	0.93	2.74	3.5436 (16)	145
C20—H20*C*⋯*Cg*2^iii^	0.96	2.81	3.7562 (17)	167
C26—H26⋯*Cg*3^iii^	0.93	2.89	3.7700 (17)	158

Symmetry codes: (i) 
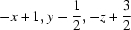
; (ii) 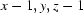; (iii) 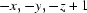.

**Fig. 1.**
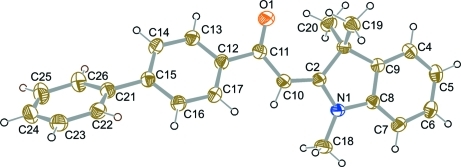
The molecular structure of the title compound. Displacement ellipsoids for non-H atom are drawn at the 30% of probability level.

**Fig. 2.**
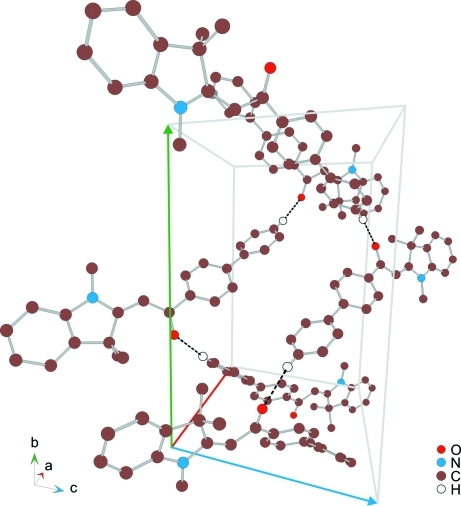
The crystal packing of the title compound viewed down the a axis, showing the molecules intermolecularly connected by weak O—H···O interactions (dashed lines). H atoms not involved in hydrogen bonding have been omitted.

## supplementary crystallographic information

### Comment

The compound 2,3-dihydro-1*H*-1,3,3-trimethyl-2-methyleneindole, well known as the Fischer base, has been widely used in organic synthesis as precursor of different types of chemical switches (Minkin, 2004; Przhiyalgovskaya *et al.*, 1987). Products with different skills to work as switches have been used in optoelectronics (Shimkin *et al.*, 2006), as organic electroluminescent materials (Cui & Kim, 2004) and dyes (Song *et al.*, 2005). Moreover, the enaminoketone derivatives of the Fischer base, like the title compound, are important intermediates for the synthesis of spiropyrans and spirooxazines (Corns *et al.*, 2009), as well as for the preparation of acetylenic products *via* enaminoketone fragmentation (Tarshits *et al.*, 2005).

The structure of the title compound [alternative name: (*E*)-2,3-dihydro-2-(biphenylacylidene)-1,3,3-trimethyl-1*H*-indol], C_25_H_23_NO, has monoclinic (*P*2_1_/*c*) symmetry. The crystal structure exhibits C—H···O intramolecular contacts in a bifurcated fashion with C19 and C20 as the donor atoms and the O atom of carbonyl group O1 as the acceptor (Table 1, Fig. 1). Moreover, despite the molecule presents high conjugation degree, the molecule is not planar; the benzene group (C12···C17) is rotated with respect to indole group (N1···C9) with the dihedral angle of 33.19 (5)°, while the biphenyl rings presents rotation showed by the dihedral angle of 37.13 (5)°.

The intermolecular assembly presents an antiparallel arrangement in a head-to-tail stacking mode especially favored by edge-to-face weak C—H···π interaction between 2.739 to 2.891 Å (see Table 2). Moreover, the weak C—H···O interaction formed by C24–H24C···O1 propagates in the *ac* plane (Fig. 2).

### Experimental

A mixture of 4-biphenylcarboxylic acid (9.59 g, 48.38 mmol) and thionyl chloride (1.5 eq., 8.63 g, 72.57 mmol) in 24 ml of dry benzene was refluxed for 1.5 h, afterward the solvent and the excess of thionyl chloride was evaporated under vacuum. For complete removal of the thionyl chloride, 24 ml of petroleum ether was added to the residue and then eliminated under vacuum. The 4-biphenyl-carbonyl chloride thus obtained was dissolved in 60 ml of dry benzene, then added to a mixture of the Fischer base 1,3,3-trimethyl-2-methyleneindoline (1 eq., 8.38 g, 48.38 mmol) and triethylamine (1.2 eq., 5.81 g, 58.04 mmol) in 60 ml of dry benzene. The reaction mixture was maintained at 40 °C for 2 h, after that it was allowed to stand overnight at room temperature. The final reaction mixture was first washed with water and the organic phase was separated and removed under vacuum. The resulting solid was washed successively with isopropyl alcohol. The crude product was purified using a column chromatography with chloroform as a mobile phase. Suitable crystals for X-ray diffraction were obtained from toluene by slow evaporation.

### Refinement

The positional parameters of H atoms were calculated geometrically (C—H = 0.93 Å for aromatic CH and 0.96 Å for methyl CH_3_). The displacement parameters for H atoms were fixed as *U*_iso_(H) = 1.2*U*_eq_(carrier atom) or *U*_iso_(H) = 1.5*U*_eq_(carrier atom).

### Crystal data

**Table:**

C_25_H_23_NO	*F*(000) = 752
*M**_r_* = 353.44	*D*_x_ = 1.203 Mg m^−^^3^
Monoclinic, *P*2_1_/*c*	Mo *K*α radiation, λ = 0.71073 Å
Hall symbol: -P 2ybc	Cell parameters from 7167 reflections
*a* = 12.3274 (13) Å	θ = 2.1–25.3°
*b* = 15.7586 (16) Å	µ = 0.07 mm^−^^1^
*c* = 10.3848 (11) Å	*T* = 298 K
β = 104.719 (2)°	Prism, yellow
*V* = 1951.2 (4) Å^3^	0.36 × 0.28 × 0.26 mm
*Z* = 4	

### Data collection

**Table:**

Bruker SMART APEX CCD area-detector diffractometer	2585 reflections with *I* > 2σ(*I*)
Radiation source: fine-focus sealed tube	*R*_int_ = 0.033
graphite	θ_max_ = 25.4°, θ_min_ = 1.7°
Detector resolution: 0.83 pixels mm^-1^	*h* = −14→14
ω scans	*k* = −18→19
15883 measured reflections	*l* = −12→12
3584 independent reflections	

### Refinement

**Table:**

Refinement on *F*^2^	Primary atom site location: structure-invariant direct methods
Least-squares matrix: full	Secondary atom site location: difference Fourier map
*R*[*F*^2^ > 2σ(*F*^2^)] = 0.038	Hydrogen site location: inferred from neighbouring sites
*wR*(*F*^2^) = 0.106	H-atom parameters constrained
*S* = 0.97	*w* = 1/[σ^2^(*F*_o_^2^) + (0.065*P*)^2^] where *P* = (*F*_o_^2^ + 2*F*_c_^2^)/3
3584 reflections	(Δ/σ)_max_ < 0.001
247 parameters	Δρ_max_ = 0.18 e Å^−^^3^
0 restraints	Δρ_min_ = −0.18 e Å^−^^3^
0 constraints	

### Fractional atomic coordinates and isotropic or equivalent isotropic displacement parameters (Å^2^)

**Table:**

	*x*	*y*	*z*	*U*_iso_*/*U*_eq_	
O1	0.11145 (9)	0.15214 (6)	0.45956 (10)	0.0720 (3)	
N1	−0.10316 (9)	0.01690 (6)	0.12145 (10)	0.0495 (3)	
C2	−0.04032 (10)	0.06729 (8)	0.22033 (12)	0.0443 (3)	
C3	−0.09068 (10)	0.15640 (7)	0.20258 (12)	0.0429 (3)	
C4	−0.26386 (11)	0.20310 (9)	0.01194 (13)	0.0520 (3)	
H4	−0.2620	0.2591	0.0405	0.062*	
C5	−0.34517 (11)	0.17673 (10)	−0.09918 (14)	0.0577 (4)	
H5	−0.3978	0.2153	−0.1458	0.069*	
C6	−0.34813 (11)	0.09381 (10)	−0.14058 (14)	0.0579 (4)	
H6	−0.4032	0.0770	−0.2153	0.069*	
C7	−0.27119 (12)	0.03476 (9)	−0.07375 (13)	0.0555 (4)	
H7	−0.2736	−0.0214	−0.1017	0.067*	
C8	−0.19038 (10)	0.06262 (8)	0.03644 (12)	0.0447 (3)	
C9	−0.18599 (10)	0.14580 (8)	0.07967 (12)	0.0424 (3)	
C10	0.04982 (11)	0.03623 (8)	0.31302 (13)	0.0527 (4)	
H10	0.0659	−0.0207	0.3034	0.063*	
C11	0.12365 (11)	0.07890 (9)	0.42363 (13)	0.0523 (4)	
C12	0.22233 (11)	0.02928 (8)	0.50225 (13)	0.0472 (3)	
C13	0.26762 (11)	0.04889 (9)	0.63521 (13)	0.0530 (4)	
H13	0.2360	0.0924	0.6741	0.064*	
C14	0.35843 (11)	0.00524 (9)	0.71096 (13)	0.0528 (4)	
H14	0.3854	0.0185	0.8007	0.063*	
C15	0.41041 (10)	−0.05831 (8)	0.65561 (12)	0.0447 (3)	
C16	0.36776 (10)	−0.07546 (8)	0.52088 (13)	0.0490 (3)	
H16	0.4028	−0.1160	0.4802	0.059*	
C17	0.27469 (11)	−0.03366 (8)	0.44639 (13)	0.0504 (3)	
H17	0.2465	−0.0478	0.3572	0.060*	
C18	−0.08214 (14)	−0.07245 (9)	0.10425 (17)	0.0717 (5)	
H18A	−0.0069	−0.0797	0.0960	0.107*	
H18B	−0.1341	−0.0931	0.0253	0.107*	
H18C	−0.0916	−0.1036	0.1801	0.107*	
C19	−0.00644 (11)	0.22263 (9)	0.17900 (15)	0.0586 (4)	
H19A	0.0231	0.2048	0.1063	0.088*	
H19B	0.0537	0.2282	0.2580	0.088*	
H19C	−0.0435	0.2763	0.1579	0.088*	
C20	−0.13763 (13)	0.18132 (10)	0.32054 (13)	0.0621 (4)	
H20A	−0.1736	0.2357	0.3036	0.093*	
H20B	−0.0775	0.1843	0.4000	0.093*	
H20C	−0.1912	0.1396	0.3319	0.093*	
C21	0.50510 (10)	−0.10866 (8)	0.73665 (13)	0.0483 (3)	
C22	0.59153 (11)	−0.13759 (9)	0.68482 (15)	0.0555 (4)	
H22	0.5925	−0.1234	0.5982	0.067*	
C23	0.67627 (12)	−0.18739 (9)	0.76088 (17)	0.0687 (5)	
H23	0.7339	−0.2061	0.7251	0.082*	
C24	0.67611 (15)	−0.20941 (10)	0.88879 (19)	0.0773 (5)	
H24	0.7326	−0.2437	0.9391	0.093*	
C25	0.59186 (15)	−0.18033 (11)	0.94158 (17)	0.0782 (5)	
H25	0.5919	−0.1944	1.0286	0.094*	
C26	0.50713 (12)	−0.13039 (10)	0.86699 (15)	0.0640 (4)	
H26	0.4507	−0.1110	0.9043	0.077*	

### Atomic displacement parameters (Å^2^)

**Table:**

	*U*^11^	*U*^22^	*U*^33^	*U*^12^	*U*^13^	*U*^23^
O1	0.0676 (7)	0.0569 (7)	0.0752 (7)	0.0108 (5)	−0.0118 (6)	−0.0149 (5)
N1	0.0500 (6)	0.0393 (6)	0.0520 (6)	0.0035 (5)	−0.0001 (5)	−0.0079 (5)
C2	0.0436 (7)	0.0426 (7)	0.0450 (7)	−0.0015 (6)	0.0081 (6)	−0.0031 (6)
C3	0.0419 (7)	0.0388 (7)	0.0466 (7)	0.0005 (5)	0.0089 (6)	−0.0039 (6)
C4	0.0483 (8)	0.0471 (8)	0.0605 (9)	0.0028 (6)	0.0138 (7)	0.0052 (6)
C5	0.0444 (8)	0.0685 (10)	0.0574 (9)	0.0055 (7)	0.0075 (7)	0.0158 (8)
C6	0.0464 (8)	0.0744 (11)	0.0476 (8)	−0.0038 (7)	0.0024 (6)	0.0030 (7)
C7	0.0546 (8)	0.0565 (8)	0.0512 (8)	−0.0033 (7)	0.0058 (7)	−0.0082 (7)
C8	0.0421 (7)	0.0478 (8)	0.0433 (7)	0.0000 (6)	0.0092 (6)	−0.0010 (6)
C9	0.0403 (7)	0.0438 (7)	0.0436 (7)	−0.0009 (5)	0.0116 (6)	0.0013 (6)
C10	0.0513 (8)	0.0421 (7)	0.0575 (9)	0.0042 (6)	0.0004 (7)	−0.0033 (6)
C11	0.0502 (8)	0.0483 (8)	0.0536 (8)	0.0014 (6)	0.0044 (7)	−0.0017 (7)
C12	0.0445 (7)	0.0486 (8)	0.0454 (7)	−0.0028 (6)	0.0058 (6)	0.0012 (6)
C13	0.0490 (8)	0.0539 (8)	0.0535 (8)	0.0030 (6)	0.0080 (7)	−0.0059 (6)
C14	0.0498 (8)	0.0616 (9)	0.0428 (7)	−0.0012 (7)	0.0040 (6)	−0.0036 (7)
C15	0.0385 (7)	0.0461 (7)	0.0476 (7)	−0.0050 (6)	0.0074 (6)	0.0036 (6)
C16	0.0455 (7)	0.0496 (8)	0.0512 (8)	0.0011 (6)	0.0111 (6)	−0.0014 (6)
C17	0.0503 (8)	0.0555 (8)	0.0419 (7)	−0.0009 (6)	0.0052 (6)	−0.0018 (6)
C18	0.0715 (10)	0.0470 (9)	0.0850 (11)	0.0077 (7)	−0.0012 (9)	−0.0164 (8)
C19	0.0524 (8)	0.0474 (8)	0.0721 (10)	−0.0047 (6)	0.0088 (7)	0.0017 (7)
C20	0.0611 (9)	0.0707 (10)	0.0547 (9)	0.0088 (8)	0.0151 (7)	−0.0113 (7)
C21	0.0423 (7)	0.0448 (7)	0.0542 (8)	−0.0062 (6)	0.0059 (6)	0.0023 (6)
C22	0.0464 (8)	0.0543 (8)	0.0618 (9)	−0.0025 (6)	0.0061 (7)	−0.0063 (7)
C23	0.0493 (9)	0.0616 (10)	0.0860 (12)	0.0067 (7)	0.0001 (8)	−0.0153 (9)
C24	0.0683 (11)	0.0556 (10)	0.0896 (13)	0.0104 (8)	−0.0137 (10)	0.0073 (9)
C25	0.0781 (12)	0.0776 (11)	0.0711 (11)	0.0085 (10)	0.0042 (9)	0.0251 (9)
C26	0.0575 (9)	0.0707 (10)	0.0617 (10)	0.0040 (7)	0.0114 (8)	0.0175 (8)

### Geometric parameters (Å, °)

**Table:**

O1—C11	1.2340 (16)	C14—H14	0.9300
N1—C2	1.3714 (15)	C15—C16	1.3904 (18)
N1—C8	1.4043 (15)	C15—C21	1.4836 (17)
N1—C18	1.4509 (17)	C16—C17	1.3775 (17)
C2—C10	1.3628 (17)	C16—H16	0.9300
C2—C3	1.5276 (17)	C17—H17	0.9300
C3—C9	1.5085 (17)	C18—H18A	0.9600
C3—C20	1.5329 (18)	C18—H18B	0.9600
C3—C19	1.5352 (18)	C18—H18C	0.9600
C4—C9	1.3742 (17)	C19—H19A	0.9600
C4—C5	1.3861 (19)	C19—H19B	0.9600
C4—H4	0.9300	C19—H19C	0.9600
C5—C6	1.373 (2)	C20—H20A	0.9600
C5—H5	0.9300	C20—H20B	0.9600
C6—C7	1.3830 (19)	C20—H20C	0.9600
C6—H6	0.9300	C21—C22	1.3871 (19)
C7—C8	1.3840 (17)	C21—C26	1.3902 (19)
C7—H7	0.9300	C22—C23	1.3826 (19)
C8—C9	1.3821 (18)	C22—H22	0.9300
C10—C11	1.4383 (17)	C23—C24	1.373 (2)
C10—H10	0.9300	C23—H23	0.9300
C11—C12	1.5010 (18)	C24—C25	1.371 (2)
C12—C13	1.3864 (17)	C24—H24	0.9300
C12—C17	1.3883 (18)	C25—C26	1.378 (2)
C13—C14	1.3775 (18)	C25—H25	0.9300
C13—H13	0.9300	C26—H26	0.9300
C14—C15	1.3897 (19)		
			
C2—N1—C8	111.73 (10)	C14—C15—C16	117.39 (12)
C2—N1—C18	124.71 (11)	C14—C15—C21	121.99 (12)
C8—N1—C18	123.55 (11)	C16—C15—C21	120.58 (12)
C10—C2—N1	121.67 (12)	C17—C16—C15	121.35 (13)
C10—C2—C3	130.46 (11)	C17—C16—H16	119.3
N1—C2—C3	107.87 (10)	C15—C16—H16	119.3
C9—C3—C2	101.86 (9)	C16—C17—C12	121.08 (12)
C9—C3—C20	109.48 (10)	C16—C17—H17	119.5
C2—C3—C20	111.31 (11)	C12—C17—H17	119.5
C9—C3—C19	110.69 (11)	N1—C18—H18A	109.5
C2—C3—C19	111.94 (11)	N1—C18—H18B	109.5
C20—C3—C19	111.18 (11)	H18A—C18—H18B	109.5
C9—C4—C5	119.50 (13)	N1—C18—H18C	109.5
C9—C4—H4	120.2	H18A—C18—H18C	109.5
C5—C4—H4	120.2	H18B—C18—H18C	109.5
C6—C5—C4	120.13 (13)	C3—C19—H19A	109.5
C6—C5—H5	119.9	C3—C19—H19B	109.5
C4—C5—H5	119.9	H19A—C19—H19B	109.5
C5—C6—C7	121.52 (13)	C3—C19—H19C	109.5
C5—C6—H6	119.2	H19A—C19—H19C	109.5
C7—C6—H6	119.2	H19B—C19—H19C	109.5
C6—C7—C8	117.34 (13)	C3—C20—H20A	109.5
C6—C7—H7	121.3	C3—C20—H20B	109.5
C8—C7—H7	121.3	H20A—C20—H20B	109.5
C9—C8—C7	122.01 (12)	C3—C20—H20C	109.5
C9—C8—N1	108.77 (10)	H20A—C20—H20C	109.5
C7—C8—N1	129.22 (12)	H20B—C20—H20C	109.5
C4—C9—C8	119.49 (12)	C22—C21—C26	118.11 (13)
C4—C9—C3	130.76 (12)	C22—C21—C15	121.79 (12)
C8—C9—C3	109.74 (10)	C26—C21—C15	120.07 (13)
C2—C10—C11	129.28 (13)	C23—C22—C21	120.55 (14)
C2—C10—H10	115.4	C23—C22—H22	119.7
C11—C10—H10	115.4	C21—C22—H22	119.7
O1—C11—C10	125.30 (12)	C24—C23—C22	120.63 (16)
O1—C11—C12	117.83 (12)	C24—C23—H23	119.7
C10—C11—C12	116.86 (12)	C22—C23—H23	119.7
C13—C12—C17	117.59 (12)	C25—C24—C23	119.32 (15)
C13—C12—C11	119.42 (12)	C25—C24—H24	120.3
C17—C12—C11	122.92 (12)	C23—C24—H24	120.3
C14—C13—C12	121.37 (13)	C24—C25—C26	120.61 (16)
C14—C13—H13	119.3	C24—C25—H25	119.7
C12—C13—H13	119.3	C26—C25—H25	119.7
C13—C14—C15	121.13 (12)	C25—C26—C21	120.77 (15)
C13—C14—H14	119.4	C25—C26—H26	119.6
C15—C14—H14	119.4	C21—C26—H26	119.6
			
C8—N1—C2—C10	−179.03 (12)	N1—C2—C10—C11	−179.06 (13)
C18—N1—C2—C10	0.1 (2)	C3—C2—C10—C11	1.0 (2)
C8—N1—C2—C3	0.94 (14)	C2—C10—C11—O1	6.0 (2)
C18—N1—C2—C3	−179.94 (13)	C2—C10—C11—C12	−174.96 (14)
C10—C2—C3—C9	178.67 (14)	O1—C11—C12—C13	26.4 (2)
N1—C2—C3—C9	−1.30 (13)	C10—C11—C12—C13	−152.73 (13)
C10—C2—C3—C20	−64.73 (18)	O1—C11—C12—C17	−150.53 (14)
N1—C2—C3—C20	115.30 (12)	C10—C11—C12—C17	30.35 (19)
C10—C2—C3—C19	60.39 (18)	C17—C12—C13—C14	−2.6 (2)
N1—C2—C3—C19	−119.58 (12)	C11—C12—C13—C14	−179.74 (12)
C9—C4—C5—C6	0.4 (2)	C12—C13—C14—C15	2.2 (2)
C4—C5—C6—C7	0.0 (2)	C13—C14—C15—C16	0.5 (2)
C5—C6—C7—C8	−0.3 (2)	C13—C14—C15—C21	−177.26 (12)
C6—C7—C8—C9	0.35 (19)	C14—C15—C16—C17	−2.71 (19)
C6—C7—C8—N1	−179.92 (13)	C21—C15—C16—C17	175.13 (12)
C2—N1—C8—C9	−0.12 (15)	C15—C16—C17—C12	2.2 (2)
C18—N1—C8—C9	−179.25 (13)	C13—C12—C17—C16	0.5 (2)
C2—N1—C8—C7	−179.88 (12)	C11—C12—C17—C16	177.46 (12)
C18—N1—C8—C7	1.0 (2)	C14—C15—C21—C22	−146.14 (14)
C5—C4—C9—C8	−0.41 (19)	C16—C15—C21—C22	36.12 (18)
C5—C4—C9—C3	−179.14 (12)	C14—C15—C21—C26	35.92 (18)
C7—C8—C9—C4	0.03 (19)	C16—C15—C21—C26	−141.81 (14)
N1—C8—C9—C4	−179.75 (11)	C26—C21—C22—C23	0.7 (2)
C7—C8—C9—C3	179.01 (12)	C15—C21—C22—C23	−177.27 (12)
N1—C8—C9—C3	−0.77 (14)	C21—C22—C23—C24	0.3 (2)
C2—C3—C9—C4	−179.92 (13)	C22—C23—C24—C25	−1.1 (2)
C20—C3—C9—C4	62.16 (18)	C23—C24—C25—C26	0.9 (3)
C19—C3—C9—C4	−60.75 (18)	C24—C25—C26—C21	0.2 (2)
C2—C3—C9—C8	1.25 (13)	C22—C21—C26—C25	−0.9 (2)
C20—C3—C9—C8	−116.67 (12)	C15—C21—C26—C25	177.07 (14)
C19—C3—C9—C8	120.42 (12)		

### Hydrogen-bond geometry (Å, °)

**Table:**

Cg1, Cg2 and Cg3 are the centroids of the C21–C26, C12–C17 and C4–C9 rings, respectively.

**Table:**

*D*—H···*A*	*D*—H	H···*A*	*D*···*A*	*D*—H···*A*
C19—H19B···O1	0.96	2.36	3.112 (2)	135.
C20—H20B···O1	0.96	2.31	3.078 (2)	137.
C24—H24···O1^i^	0.93	2.54	3.465 (2)	171.
C7—H7···Cg1^ii^	0.93	2.74	3.5436 (16)	145
C20—H20C···Cg2^iii^	0.96	2.81	3.7562 (17)	167
C26—H26···Cg3^iii^	0.93	2.89	3.7700 (17)	158

Symmetry codes: (i) −*x*+1, *y*−1/2, −*z*+3/2; (ii) *x*−1, *y*, *z*−1; (iii) −*x*, −*y*, −*z*+1.
